# A comparison of GABA-ergic (propofol) and non-GABA-ergic (dexmedetomidine) sedation on visual and motor cortical oscillations, using magnetoencephalography

**DOI:** 10.1016/j.neuroimage.2021.118659

**Published:** 2021-12-15

**Authors:** Neeraj Saxena, Suresh D. Muthukumaraswamy, Lewys Richmond, Adele Babic, Krish D. Singh, Judith E. Hall, Richard G. Wise, Alexander D. Shaw

**Affiliations:** a Cardiff University Brain Research Imaging Centre (CUBRIC), School of Psychology, Cardiff University, Cardiff CF24 4HQ, United Kingdom; b Department of Anaesthetics, Intensive Care and Pain Medicine, Cwm Taf Morgannwg University Health Board, Llantrisant CF72 8XR, United Kingdom; c School of Pharmacy, Faculty of Medical and Health Sciences, Auckland University, Auckland 1123, New Zealand; d School of Psychology, Faculty of Medical and Health Sciences, Auckland University, Auckland 1123, New Zealand; e Department of Anaesthetics, Morriston Hospital, Swansea, SA6 6NL, United Kingdom; f Department of Anaesthetics, Royal Gwent Hospital, Newport, NP20 2UB, United Kingdom; g Department of Anaesthetics, Intensive Care and Pain Medicine, School of Medicine, Cardiff University, Cardiff CF14 4XW, United Kingdom; h Institute for Advanced Biomedical Technologies, "G. D'Annunzio University" of Chieti-Pescara, 66100, Chieti, Italy; i Department of Neuroscience, Imaging and Clinical Sciences, "G. D'Annunzio University" of Chieti-Pescara, 66100, Chieti, Italy; j Department of Psychology, University of Exeter, United Kingdom

**Keywords:** Magnetoencephalography, Oscillations, Neurophysiology, Propofol, Dexmedetomidine, Conscious sedation

## Abstract

Studying changes in cortical oscillations can help elucidate the mechanistic link between receptor physiology and the clinical effects of anaesthetic drugs. Propofol, a GABA-ergic drug produces divergent effects on visual cortical activity: increasing induced gamma-band responses (GBR) while decreasing evoked responses. Dexmedetomidine, an α2- adrenergic agonist, differs from GABA-ergic sedatives both mechanistically and clinically as it allows easy arousability from deep sedation with less cognitive side-effects. Here we use magnetoencephalography (MEG) to characterize and compare the effects of GABA-ergic (propofol) and non-GABA-ergic (dexmedetomidine) sedation, on visual and motor cortical oscillations. Sixteen male participants received target-controlled infusions of propofol and dexmedetomidine, producing mild-sedation, in a placebo-controlled, cross-over study. MEG data was collected during a combined visuomotor task. The key findings were that propofol significantly enhanced visual stimulus induced GBR (44% increase in amplitude) while dexmedetomidine decreased it (40%). Propofol also decreased the amplitudes of the Mv100 (visual M100) (27%) and Mv150 (52%) visual evoked fields (VEF), whilst dexmedetomidine had no effect on these. During the motor task, neither drug had any significant effect on movement related gamma synchrony (MRGS), movement related beta de-synchronisation (MRBD) or Mm100 (movement-related M100) movement-related evoked fields (MEF), although dexmedetomidine slowed the Mm300. Dexmedetomidine increased (92%) post-movement beta synchronisation/rebound (PMBR) power while propofol reduced it (70%, statistically non- significant). Overall, dexmedetomidine and propofol, at equi-sedative doses, produce contrasting effects on visual induced GBR, VEF, PMBR and MEF. These findings provide a mechanistic link between the known receptor physiology of these sedative drugs with their known clinical effects and may be used to explore mechanisms of other anaesthetic drugs on human consciousness.

## 1 Introduction

Our understanding of the mechanisms of anaesthesia and the neural correlates of anaesthesia-induced unconsciousness is incomplete. A range of theories of anaesthetic mechanism point towards a breakdown of communication between key brain regions as a common endpoint in anaesthesia related unconsciousness (Mashour, 2013). Oscillatory synchronisation in different frequency bands contributes to long-range neural communication. Of these oscillations, those in the high frequency band (gamma band (30–80 Hz)) are considered key for information processing in the brain (Buzsaki and Wang, 2012). Studying changes in these neural oscillations, both task-related and at rest, provides an opportunity to explore the systems-level mechanistic underpinnings of anaesthetic drug effects and to link them with their known receptor-level effects.

In response to a sustained simple visual contrast pattern, sustained narrow band gamma band oscillations are generated in the visual cortex. These patterns arise from the interactions between the excitatory and inhibitory neural networks, which shape both the amplitude and peak frequency of these gamma oscillations. According to the pyramidal-interneuron gamma (PING) model, the local interaction of superficial pyramidal cells and inhibitory interneuron populations underlies oscillations in the gamma-frequency band (30+ Hz) (Whittington et al., 1997). The validity of the proposed PING model has been demonstrated in human visual gamma responses. Applying dynamic causal modelling it has been shown that the frequency and amplitude of visual gamma oscillations is determined by the interactions between the pyramidal cells and inhibitory interneurons in the canonical PING circuit and is modulated in humans after receiving the GABA reuptake inhibitor tiagabine (Shaw et al., 2017). Similar to visual cortex oscillations, modelling for the human motor cortex oscillations (M1) has shown the best fitting model to be one involving an interacting canonical micro-circuit including pyramidal and inhibitory interneuron circuits (Bhatt et al., 2016). We have previously demonstrated that sedation with propofol (as a representative drug with primarily GABA-ergic action) results in increased gamma band response/power (GBR), increased alpha power suppression, and a decrease in the amplitude of the stimulus- onset evoked (both transient gamma band and visual evoked fields (VEF)) response (Saxena et al., 2013). This provided an insight into the possible separation of the neural generators of visual gamma oscillations (Castelo-Branco et al., 1998) and the differential effects of propofol on those generating mechanisms. Propofol appeared to inhibit thalamo-cortical pathways resulting in decreased evoked visual responses while its intracortical GABAergic inhibition resulted in an enhanced induced gamma amplitude. This mechanistic discovery provides a potential biomarker to study and refine different pharmacological compounds that have similar clinical actions.

Dexmedetomidine produces sedation through mechanisms distinct from the commonly used GABAergic anaesthetic drugs (e.g., propofol and midazolam). Dexmedetomidine selectively acts on the α2- adrenergic receptors of the locus coeruleus, projecting to the preoptic area, which activates the inhibitory outputs to the arousal centres and results in sedation (Nelson et al., 2003). Dexmedetomidine's neurophysiological mechanisms, replicating ‘restorative sleep’ through activity on brainstem and normal sleep pathways, instead of the cortical suppression seen with GABAergic sedatives, may make it clinically advantageous especially in critically ill patients requiring long-term sedation (Riker et al., 2009).

In this experiment we used MEG to characterise and compare the effects of propofol and dexmedetomidine on task-based (visual and motor) cortical oscillations in a placebo-controlled, cross-over, single-blind study. Comparing the system-level effects of these two drugs, with distinct receptor-level mechanisms, will help understand the commonalities and differences in the pathways resulting in similar behavioural outcomes (i.e., mild sedation). Based on the current understanding of dexmedetomidine's actions, i.e., primarily at the locus coeruleus, leading on to the suppression of the cortex, we expected it to suppress thalamocortical responses, with suppression of cortical activity. Unlike dexmedetomidine, propofol is likely to produce a marked suppression of thalamocortical activity, and also a marked (direct) inhibition of cortical activity due to its direct activity at widespread GABA receptors. We, therefore, hypothesised that unlike propofol, dexmedetomidine will cause a reduction in visual induced GBR, while propofol causes an increase in induced GBR. We also hypothesised that the visual evoked field (VEF) changes will be greater with propofol than with dexmedetomidine.

In addition, we aimed to characterise the effects of propofol and dexmedetomidine on motor cortical oscillatory activity during a simple finger abduction task and movement-related evoked fields (MEF). Previous work (Campbell et al., 2014; Hall et al., 2011; Muthukumaraswamy et al., 2013) on GABA-ergic activity on motor oscillations has been inconclusive. We were unable to find any study on dexmedetomidine's effects on motor cortical oscillations. For this exploratory work, we hypothesised that motor cortex gamma activity generators would behave similarly to those of the visual cortex and contrasting effects of dexmedetomidine and propofol would be demonstrable (i.e., an increase in induced motor gamma with propofol but not with dexmedetomidine).

## 2 Materials and methods

### 2.1 Participants

Sixteen right-handed healthy male participants (mean age 27.3 years (SD 5.2, range 21–40) were recruited following a detailed screening procedure. Due to the potential of variability in the cerebral GABA levels/activity in females, dependant on the phase of their menstrual cycle and its potential to confound the GABAergic activity being studied (Harada et al., 2011; Sumner et al., 2018), we chose to limit our study population to males (similar to our previous work (Saxena et al., 2013)). The study was approved by Cardiff University's Research Ethics Committee and all participants gave informed written consent. Medical screening was performed to ensure that all participants were in good physical and mental health and not on any regular medication (American Society of Anesthesiologists physical status 1). Any volunteer with complaints of regular heartburn or hiatus hernia, known or suspected allergies to propofol or dexmedetomidine (or its constituents), regular smokers, those who snored frequently or excessively, or who had a potentially difficult-to-manage airway were excluded.

### 2.2 Monitoring, drug administration and sedation assessment

**Fig. 1:**
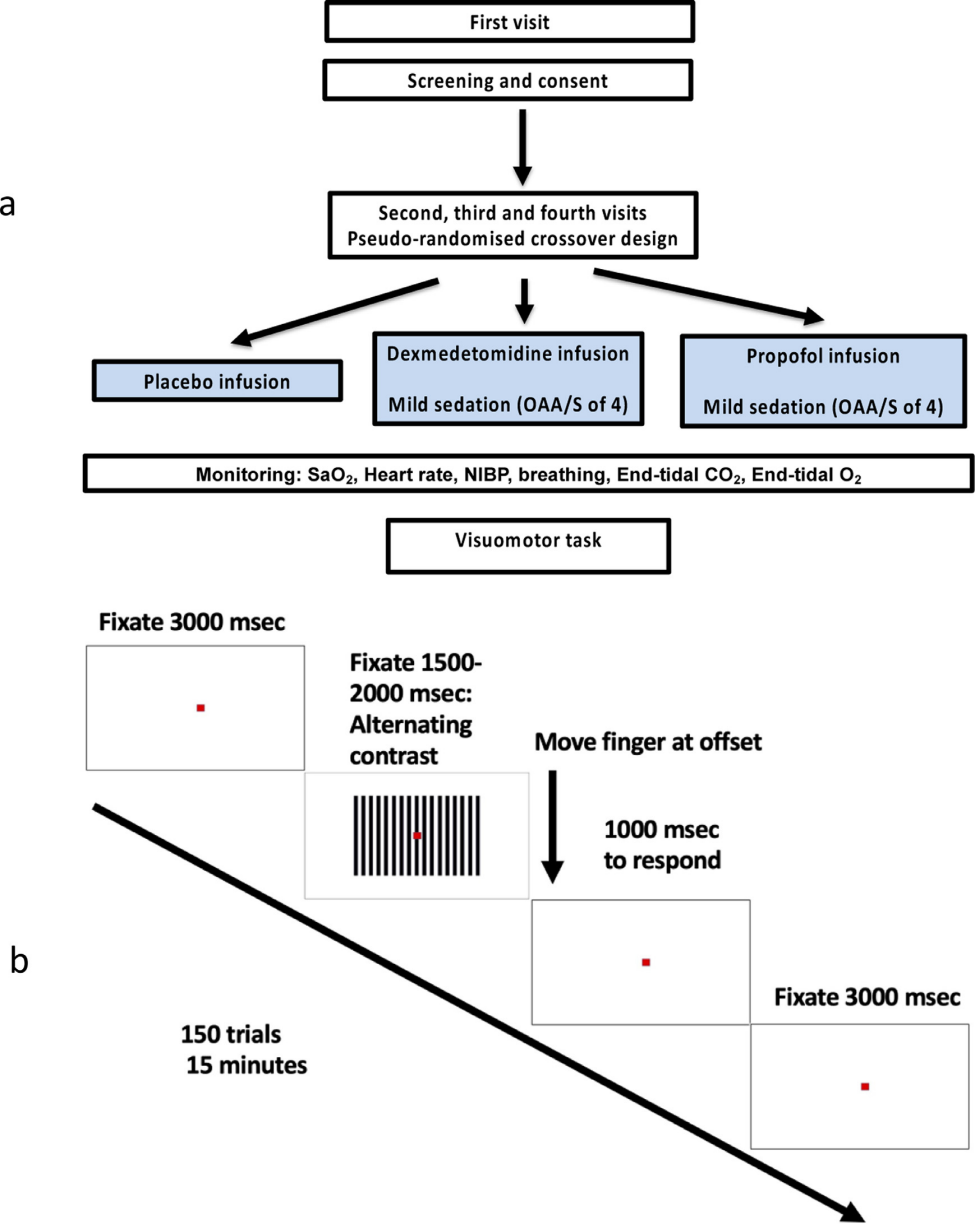
(a) Schematic of the experimental design. (**b)** Paradigm for visuomotor task. Each trial duration was about 6 s (total number of trials = 150 (75 at 100% visual contrast and 75 at 70% visual contrast).

Throughout the experiments, all participants were monitored, as per anaesthetic standards, by two anaesthetists of which one was solely involved in monitoring. Participants were instructed to follow standard pre-anaesthetic fasting guidelines. Participants received either placebo (normal saline infusion), propofol or dexmedetomidine infusion in a pseudo-randomised design (Fig. 1a). These sessions were conducted over three separate visits, with each session separated from the next by a minimum of 72 h to ensure complete clearance of the drug. For the control (normal saline) session, data were recorded starting 10 min into the infusion. Sedation level was assessed by the second anaesthetist (NS), using the modified Observer's assessment of alertness/sedation scale (OAA/S) (Thomson et al., 2009). Sedation endpoint was an OAA/S level of 4 (slurred speech with lethargic response to verbal commands).

### 2.3 Propofol administration

Propofol (Propofol-Lipuro 1%, Braun Ltd., Germany) was administered using an Asena® - PK infusion pump (Alaris Medical, UK) using a target controlled infusion based on the Marsh-pharmacokinetic model as described in our previous work (Saxena et al., 2013). While participants lay supine in the magnetically shielded room, infusion was started targeting an effect-site concentration of 0.6 mcg/ml. Once the target was reached, two minutes were allowed to ensure reliable equilibration. Drug infusion was then increased in 0.2 mcg/ml increments until the desired level of sedation was achieved.

### 2.4 Dexmedetomidine administration

Dexmedetomidine (Dexdor®, Orion Corporation, Finland) was administered using a Graseby 3500® infusion pump (Smiths Medical, UK) controlled by a personal computer using the STANPUMP software using the Dyck pharmacokinetic model (Dyck et al., 1993). Infusion was started targeting an effect site plasma concentration of 0.1 nanograms/ ml. Once the target was reached, five minutes were allowed to ensure further equilibration. Drug infusion was then increased in 0.1 nanograms/ ml increments until the desired level of sedation (OAA/S of 4) was achieved.

### 2.5 Stimulation paradigm

Once steady state sedation was achieved, participants were presented with a visual stimulus consisting of a vertical, stationary, maximum contrast, three cycles per degree, square-wave grating presented on a mean luminance background. Of a total 150 trials, 75 were displayed at maximum contrast, while the remaining 75 were displayed at 70% (‘low’) contrast. The radius of the grating was 8° of visual angle, with a continually displayed, small, central, red fixation square. The grating patch was displayed for between 1.5 and 2 s with 3 s inter-stimulus interval (displaying a fixation square only). The stimulus was presented on a projection screen controlled by Presentation®. Stimuli were displayed by a Sanyo XP41 LCD back-projection system displaying at 1024 × 768 at 60 Hz. Participants were instructed to fixate on the red square throughout the trial and perform a finger abduction at grating-offset. Activity of the first dorsal interosseous muscle during finger abduction was recorded by a bipolar EMG electrode placed either end of the muscle. Actual finger movement was recorded by an optical displacement metre (Muthukumaraswamy, 2010). Each recording session took approximately 15 min and was carried out before and during sedation (Fig. 1b).

### 2.6 MRI acquisition

All participants had a structural MRI scan either as part of the study, or as participants in previous studies in Cardiff University Brain Research Imaging Centre (CUBRIC). Scans were conducted on a GE HDx 3T MR scanner with 8 channel head coil and followed a fast spoiled gradient echo (FSPGR) sequence with 1 mm isotropic voxel resolution. Co-registration with MEG data was achieved by matching fiducial coil positions recorded in MEG to the same location on MR images.

### 2.7 MEG acquisition and analysis

Whole head MEG recordings were made using a CTF 275- channel axial gradiometer system (VSM MedTech) sampled at 1200 Hz (0–300 Hz bandpass). An additional 29 reference channels were recorded for noise cancellation purposes and the primary sensors analysed as synthetic third-order gradiometers (Vrba and Robinson, 2001). Three of the 275 channels were turned off due to excessive sensor noise. At the onset of each stimulus presentation a TTL pulse was sent to the MEG system. Participants were fitted with three electromagnetic head coils (nasion and bilateral pre-auriculars), which were localised relative to the MEG system immediately before and after the recording session. These were used for MRI/ MEG co-registration as described above.

### 2.8 MEG pre-processing

Dataset markers were placed at the initiation of finger abduction, based on a shift in the amplitude of the optical displacement metre by three standard deviations above mean noise (Cheyne et al., 2008). Where noise masked a shift corresponding to a displacement, the EMG trace from the first dorsal interosseus was used and the same algorithm applied. For the visual response, data were epoched into 4 s trials (from 2 s before to 2 s after the visual stimulus onset) to create a dataset containing only visual grating trials. For the motor response, data were epoched into 4.5 s trials consisting of 1.5 s pre- and 3 s post- finger abduction onset to create a dataset of only motor responses. Trials from both datasets were visually inspected for gross artifacts (head movements and muscle artifacts affecting a large number of sensors) and these trials were removed.

### 2.9 Visual response source localization

Visual stimulation, as used in this experiment, produces a typical response morphology: there is an initial transient broadband (50 to 100 ms) amplitude increase in the gamma frequency (40+ Hz) range followed by a longer- lasting elevation of gamma frequency amplitude in a narrower frequency range (induced response) (Muthukumaraswamy et al., 2010).

Data analysis and statistical analysis were done using custom MATLAB scripts and toolboxes. Two source localisations were performed on each dataset using synthetic aperture magnetometry; one for induced responses (SAM_ind_), and one for evoked responses (SAM_erf_) (Robinson, 2004). Correspondingly, two global covariance matrices were calculated for each dataset, one for SAM_ind_ (40–80 Hz) and one for SAM_erf_ (0–100 Hz). Based on these covariance matrices, using the beamformer algorithm (Robinson and Vrba, 1999), two sets of beamformer weights were computed for the entire brain at 4 mm isotropic voxel resolution. A local-spheres (Huang et al., 1999) volume conductor model was derived by fitting spheres to the brain surface extracted by FSL's Brain Extraction Tool (Smith, 2002).

For gamma-band SAM_ind_ imaging, virtual sensors were constructed for each beamformer voxel and student's -t images of source power changes computed using a baseline period of −1.5 to 0 s and an active period of 0 to 1.5 s. Within these images, the voxel with the strongest power increase (in the contralateral occipital lobe) was located. To reveal the time–frequency response at this peak location, the virtual sensor was repeatedly band-pass filtered between 1 and 150 Hz at 0.5 Hz frequency step intervals using an 8 Hz bandpass, 3rd order Butterworth filter (Le Van Quyen et al., 2001; Muthukumaraswamy et al., 2010). The Hilbert transform was used to obtain the amplitude envelope and spectra were computed as a percentage change from the mean pre- stimulus amplitude (−1.5 to 0 s) for each frequency band. From these spectra, the time courses of alpha (8–15 Hz) and gamma (40–80 Hz) were extracted and submitted to non-parametric permutation tests using 5000 permutations and omnibus correction for multiple corrections (Nichols and Holmes, 2002). To examine pre-stimulus amplitudes the time- frequency spectra were recomputed with no baseline correction and the average amplitudes of alpha (8–15 Hz), beta (15–40 Hz) and gamma (40–80 Hz) in the pre-stimulus period (−1.5 to 0 s) were calculated.

For SAM_erf_, the computed evoked response was passed through the 0–100 Hz beamformer weights and SAM_erf_ images (Robinson, 2004) were generated at 0.01 s intervals from 0.05 to 0.15 s. The image (usually 0.08 to 0.09 s or 0.09 to 0.1 s) with the maximal response in visual cortex was identified and the maximal voxel selected as the peak location for virtual sensor analysis. For time-domain analysis, the evoked field was computed for this virtual sensor (−0.2 to 0 s baseline, 40 Hz low-pass filter) and the peak amplitude and latency of the Mv100 (visual M100) and Mv150 responses were quantified. We also performed a spectral analysis of the evoked field using the same time-frequency techniques as above. The evoked frequency (gamma-band) response in the 0 to 0.2 s period was obtained for each condition and analysed using the same statistical methodology.

### 2.10 Motor response source localisation

The motor paradigm elicits a narrow-band response between 60 and 90 Hz (Muthukumaraswamy, 2010) termed movement- related gamma synchrony (MRGS). This paradigm also elicits a robust bilateral beta de-synchronisation (movement related beta de-synchronisation: MRBD) followed by a beta- rebound (post-movement beta synchronisation/ rebound; PMBR), more prominent in the contralateral hemisphere. Analysis of motor responses was procedurally similar to visual responses, except for the following differences. The beamforming and virtual sensor reconstruction procedure was repeated for each of these components with the beta range defined as 15–30 Hz. Guided by previous reports (Muthukumaraswamy et al., 2013) the following times were used; baseline MRGS = −1.3 to −1 s, active MRGS = 0 to 0.3 s, baseline MRBD = −1.25 s to −0.5 s, active MRBD = −0.25 s to 0.5 s, baseline PMBR = −1.25 s to −0.5 s, active PMBR = 1 to 2.5 s. Virtual sensors were created separately for each participant and each condition (pre and post, for placebo, propofol and dexmedetomidine). As per the visual analysis, time frequency content was reconstructed at the virtual sensor location with the maximal relative response. The time–frequency content of the virtual sensors was estimated by applying the Hilbert transform to estimate the amplitude content in 0.5 Hz windows between 1 and 100 Hz.

The analysis steps for MEF were similar to the VEF. Virtual sensor location generated for the motor gamma analysis was band pass filtered (1:30 Hz). Baseline period (−0.5 s to 0 s) was subtracted from post-movement (0 s- movement onset) and the peak amplitude and latency of the Mm100 (movement-related M100) were quantified and compared between groups. A window of 0.24 s to 0.40 s for Mm300 calculation.

In the first instance, for both experimental paradigms, the time–frequency spectra were estimated on the whole trial (including baseline period), with no baseline correction applied, to check for drug-induced differences in the pre- stimulus period which might confound any stimulus induced changes in frequency or amplitude. Time–frequency analysis revealed an effect of the drugs on the baseline amplitude of the visual gamma, MRGS, MRBD and PMBR sensors, hence subsequent time–frequency analysis of these sensors utilised a relative change (percentage from mean baseline) approach.

For statistical analyses, repeated-measures ANOVA was used (condition = dexmedetomidine, propofol or placebo) for condition of primary interest. Paired t-tests were used for post-hoc between-group analyses (placebo vs propofol and placebo vs dexmedetomidine). Results were corrected for multiple comparisons using Bonferroni's correction. These are presented as ‘corrected’ in the subsequent text.

## 3 Results

### 3.1 Sedation level/dose

All participants were sedated to the desired level of mild sedation (OAA/S of 4). The mean plasma concentration of propofol required was 0.83 mcg/ ml (SD 0.2 mcg/ml) and for dexmedetomidine was 0.25 ng/ml (SD 0.12 ng/ml). Both drugs reduced systolic BP (*p* < 0.005; Table 1) but had no effect on the heart rate. There was no difference in recorded head movement between the groups and there were a comparable number of trials in each group (after rejecting ‘bad’ trials, for both visual and motor analyses) (Supplementary content: Table S1).

**Table 1:** Haemodynamic changes during infusions. There was a significant decrease in systolic blood pressure (SBP) in the propofol group (* *p* = 0.017; paired *t*-test, 2- tailed) but not in diastolic blood pressure (DBP) or heart rate (HR). SD = Standard deviation.

	Dexmedetomidine		Placebo		Propofol	
	Pre-infusion	During- infusion	Pre-infusion	During- infusion	Pre-infusion	During- infusion
**SBP: Mean (SD); mm Hg**	126 ± 11	118 ± 13	123 ± 17	122 ± 11	125 ± 9	115 ± 13*
**DBP: Mean (SD); mm Hg**	70 ± 9	66 ± 7	70 ± 8	69 ± 10	70 ± 7	64 ± 10
**HR: Mean (SD); bpm**	62 ± 9	60 ± 6	63 ± 7	63 ± 7	66 ± 9	64 ± 5

### 3.2 Visual responses

**Fig. 2:**
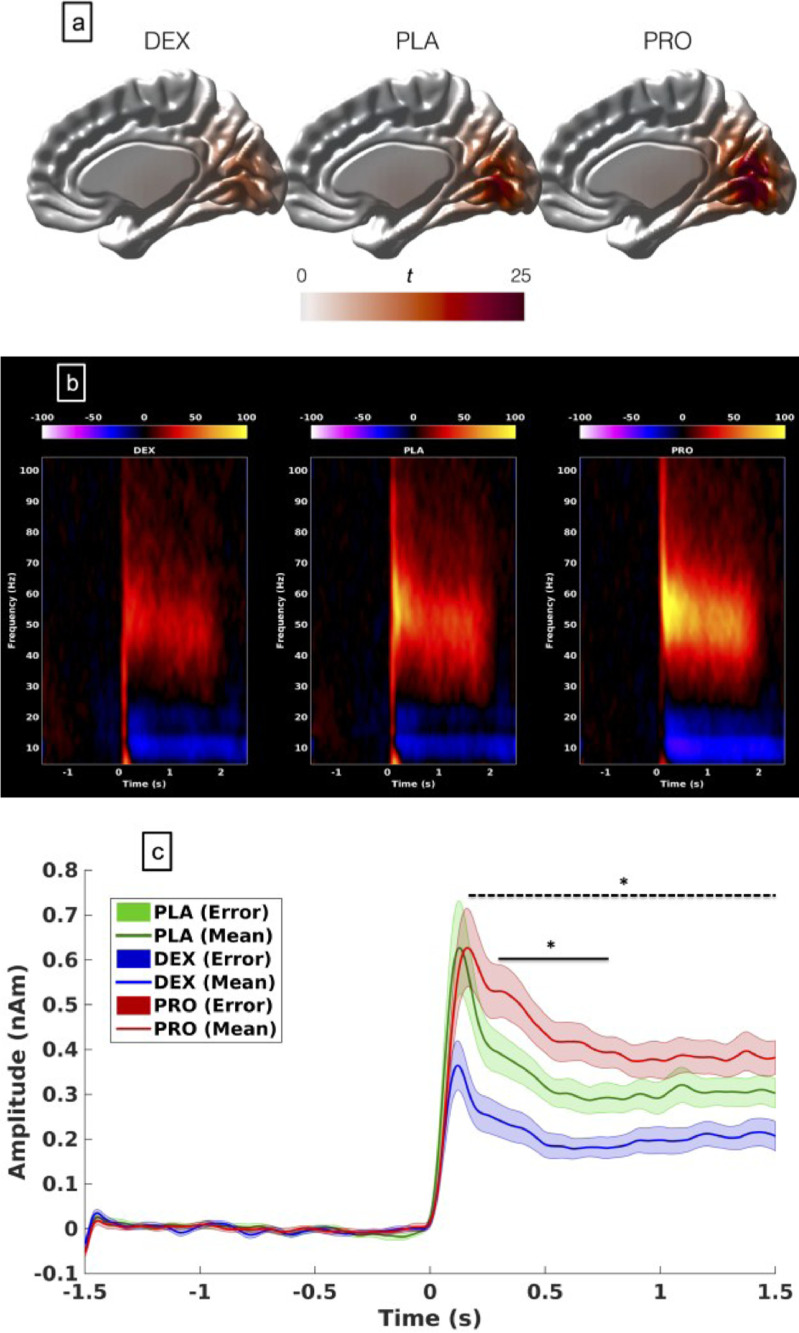
(a) Grand-averaged source localisation of gamma oscillations (40–80 Hz) for awake and sedated states. Units are t statistics. PLA = placebo, DEX = dexmedetomidine, PRO = propofol. Units are t-statistics (b): Grand-averaged time-frequency spectrograms showing source-level oscillatory amplitude (evoked + induced) changes following visual stimulation with a maximum contrast (100%) grating patch (stimulus onset at time = 0) during awake and sedated states. Spectrograms are displayed as percentage change from the pre-stimulus baseline and were computed for frequencies from 5 up to 150 Hz but truncated here to 100 Hz for visualisation purposes. (c): Envelopes of oscillatory amplitude for the gamma band (40–80 Hz). Time-periods with significant differences between the three conditions are indicated with a black bar (*p < 0.05, shaded areas represent SEM). Colour: Blue- dexmedetomidine; Green- placebo; Red- propofol: Dotted bar- difference between dexmedetomidine and placebo; Bold bar- difference between propofol and placebo (For interpretation of the references to color in this figure legend, the reader is referred to the web version of this article.).

The visual grating stimulus utilised here robustly elicits induced GBR in the primary visual cortex. The grand-averaged peak locations of the responses, in all, were located in neighbouring source reconstruction voxels (4 mm voxel size) (Fig. 2a). This analysis found similar results from both ‘maximum’ (100%) and ‘low’ (70%) contrast grating patches and therefore only the results from the maximum contrast gratings are presented here. Data from ‘low’ contrast gratings is presented in the Supplementary content (Fig. S1).

Fig. 2b shows the group visual responses. The virtual sensor reconstruction demonstrated changes in pre-stimulus gamma power between groups (F (2,30) = 3.17; *p* = 0.0035) (Supplementary content: Fig. S2) and therefore a ‘relative change’ approach was utilised for analysis. In Fig. 2c the extracted gamma (40–80 Hz) time-courses are plotted. For the high contrast stimulus, propofol resulted in a 44% increase in gamma amplitude, as compared to placebo, between 0.3–0.8 s following the stimulus (*t* = 2.73, *p* = 0.027, corrected) while dexmedetomidine resulted in a 40% decrease in amplitude between 0.1–1.5 s following the stimulus (*t* = −4.59, *p* = 0.004, corrected) (Fig. 2c). There was no change in peak-induced gamma frequency (F (2,30) = 0.074; *p* = 0.93) (Supplementary content: Fig. S3). Propofol (with high contrast gratings) resulted in an increased stimulus-induced alpha suppression by about 50% (*t* = 2.95, *p* = 0.02, corrected), however there was no change in alpha suppression by dexmedetomidine (Supplementary content: Fig. S4). There was no change in alpha suppression with either drug at low contrast settings (F (2,30) = 0.169, *p* = 0.173).

The evoked/transient gamma-band amplitude was reduced by 53% with dexmedetomidine (*t* = −3.58, *p* = 0.004, corrected) but not with propofol (*t* = 0.38, *p* = 0.7) (Supplementary content: Fig. S5a). There were no significant changes in the peak evoked/transient gamma-band frequency with the drugs (Supplementary content: Fig. S5b).

**Fig. 3:**
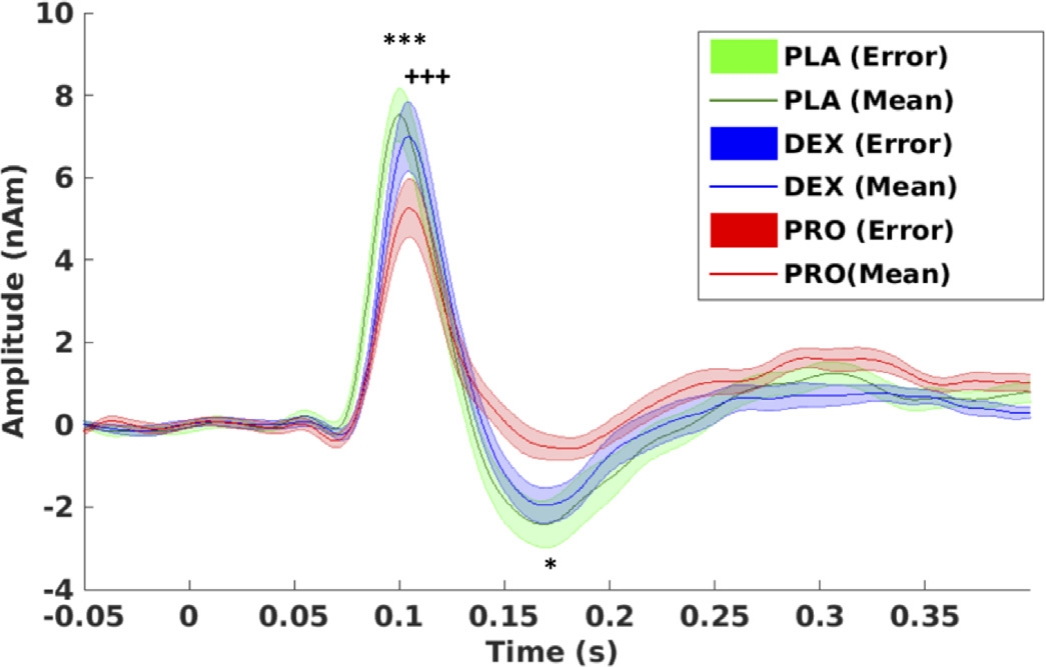
Visual responses: Source-level time-averaged evoked responses for placebo, propofol and dexmedetomidine. PLA = placebo (green), DEX = dexmedetomidine (blue), PRO = propofol (red). Significant differences were seen in Mv100 amplitudes: between propofol and placebo (***p < 0.001); Mv100 latencies: between propofol and placebo (**^+++^***p* < 0.001) and between dexmedetomidine and placebo (**^+++^***p* < 0.001); Mv150 amplitudes: between propofol and placebo (*p < 0.05). There were no significant changes between placebo and drugs on Mv150 latency. Two- tailed paired *t*-test, Bonferroni's correction applied. Bold line represents means, shaded areas represent SEM (For interpretation of the references to color in this figure legend, the reader is referred to the web version of this article.).

Fig. 3 presents the time- averaged VEF. There were significant reductions in both the amplitude of the Mv100 (mean change 27%) (*t* = 6.9, *p* < 0.001, corrected) and Mv150 (mean change 52%) (*t* = −3.0, *p* = 0.018, corrected) components during propofol sedation. However, there were no differences between placebo and dexmedetomidine (Mv100: *t* = 1.19, *p* = 0.25; Mv150: *t* = −0.89, *p* = 0.388). There was significant slowing of the Mv100 component with both propofol (*t* = −4.2, *p* < 0.001, corrected) and dexmedetomidine (*t* = −4.6, *p* < 0.001, corrected). There was however no difference between the latencies of the Mv150 component (Supplementary content: Fig. S6).

### 3.3 Motor responses

**Fig. 4:**
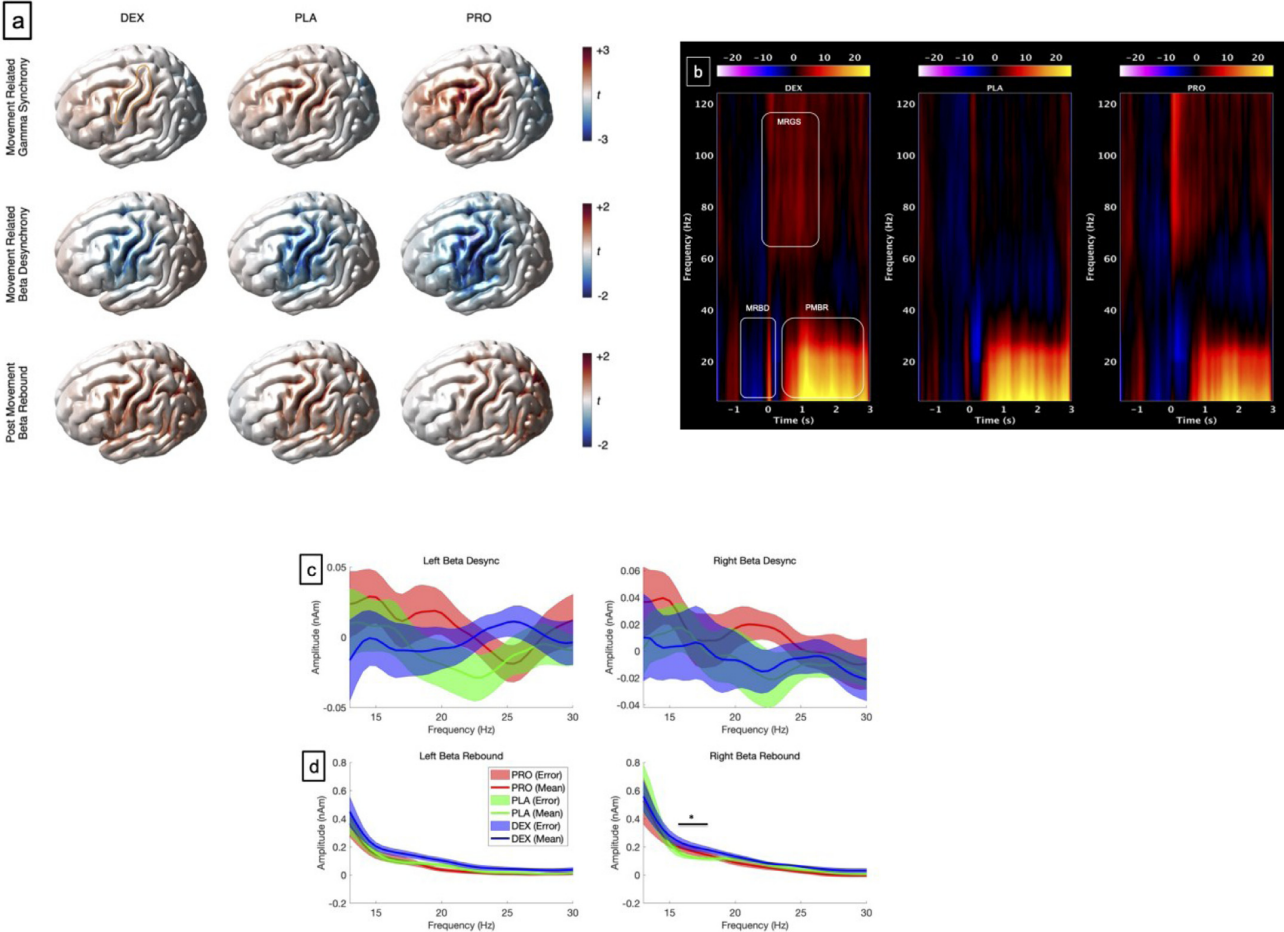
(**a)** Grand-averaged source localisation of movement related gamma and beta oscillations for awake and sedated states. Units are t statistics. (b) Grand averaged time–frequency spectrograms showing source-level oscillatory amplitude changes following movement. Spectrograms are displayed as percentage change from the pre-stimulus baseline, with amplitude depicted by heat-map colours. The rounded rectangles are only representative and not active window used for analysis (see text for active windows used). (c) Power spectra changes: Movement related beta desynchronization (MRBD): Right and left sensors. (d) Post movement beta rebound (PMBR): Right and left sensors. DEX = dexmedetomidine, PLA = placebo, PRO = propofol. PLA = placebo (green), DEX = dexmedetomidine (blue), PRO = propofol (red): Bold bar- difference between dexmedetomidine and placebo. (*p < 0.05) (For interpretation of the references to color in this figure legend, the reader is referred to the web version of this article.).

**Fig. 5:**
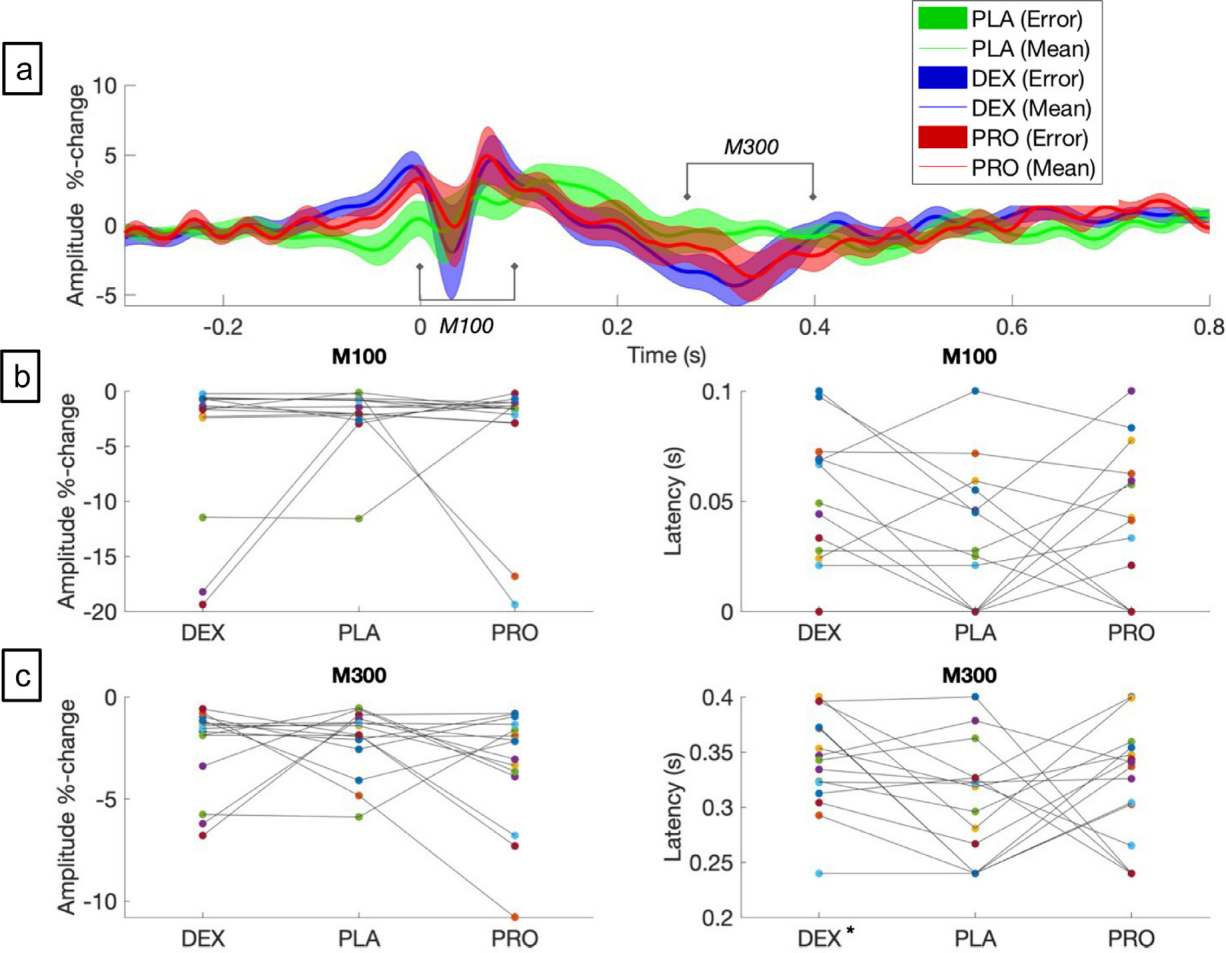
(a) Motor responses: Source-level time-averaged evoked responses for placebo, propofol and dexmedetomidine. PLA = placebo (green), DEX = dexmedetomidine (blue), PRO = propofol (red). Bold line represents means, shaded areas represent SEM. **b**) There were no significant differences between drugs, in Mm100 amplitudes or latencies. **c**) There were no significant differences between drugs, in Mm300 amplitudes. However, dexmedetomidine increased the latency as compared to placebo (*p < 0.05) (For interpretation of the references to color in this figure legend, the reader is referred to the web version of this article.).

The finger abduction task robustly elicits 3 components: a contralateral MRGS, bilateral MRBD followed by a bilateral PMBR (Fig. 4a, 4b). There were no significant changes with both drugs on the MRGS or the MRBD, either the ipsilateral (right (BD-R)) or contralateral (left (BD-L)) sides (Fig. 4c). However, PMBR revealed increased power (92%) of ipsilateral (right (BR-R)) PMBR with dexmedetomidine (between 16–18.5 Hz, *t* = 2.6, *p* = 0.044, corrected) (Fig. 4d) but not on the contralateral side (left (BR-L)). There was a non-significant reduction (70%) with propofol in the contralateral (left (BR-L)) PMBR (between 20–20.5 Hz) (*t* = −2.16, *p* = 0.1, corrected), but no change on the ipsilateral (right (BR-R)) side. There were no differences in either the amplitudes or latencies of Mm100 between the drugs against placebo (Fig. 5a–c). There were no differences in the amplitudes of Mm300 between the drugs. However, dexmedetomidine slowed the Mm300 (*t* = 2.07, *p* = 0.049, corrected) (Fig. 5a,c) compared to placebo.

## 4 Discussion

This study reports the findings from a combined visuomotor MEG paradigm recorded during a placebo-controlled, crossover, single-blind study of the sedative effects of propofol (GABA-ergic drug) and dexmedetomidine (non-GABA-ergic drug), on human cortical oscillations. The key findings were a significant increase in the visual stimulus-induced GBR with propofol (44% increase in amplitude), while there was a significant decrease with dexmedetomidine (40%). Dexmedetomidine reduced stimulus-onset-evoked GBR (53%), while propofol did not. While propofol also decreased the amplitudes of the Mv100 (27%) and Mv150 (52%) VEF, dexmedetomidine had no effect on these. Both drugs slowed Mv100 (increased latency) but had no effect on the latency of Mv150. During the motor task, neither drug had any significant effect on MRGS, MRBD or Mm100 MEF, although dexmedetomidine slowed Mm300. Dexmedetomidine also significantly increased (92%) PMBR, while propofol showed a tendency to reduce it (70%).

### 4.1 Visual oscillatory responses

#### 4.1.1 Induced and evoked GBR

Our previous results (Saxena et al., 2013), demonstrating the *in-vivo* modifiability of human GBR, were reproduced here using a more robust version of the stimulation protocol (Muthukumaraswamy et al., 2013). The dissociation between the evoked and induced responses by propofol were considered akin to the dissociation between evoked responses and induced GBR representing separate thalamocortical and intracortical mechanisms, respectively, in the generation of high frequency oscillations (Castelo-Branco et al., 1998); a finding which has also been demonstrated in human intra-cortical recordings (Privman et al., 2010). According to the PING model, GABA-ergic inhibition of interneuronal control may facilitate hypersynchronicity presenting as an increased power of the induced GBR. Suppression of both evoked responses and induced GBR by dexmedetomidine supports a depressive action on thalamocortical generators with no local intracortical facilitation, as hypothesised.

Plourde and Arseneau (2017), demonstrated that dexmedetomidine produced a dose-dependant attenuation of thalamic and cortical oscillations in the 30–200 Hz frequency bands. Dexmedetomidine attenuates both thalamic and cortical oscillations to a similar degree while propofol has a greater effect on the thalamic oscillations than cortical oscillations (Reed and Plourde, 2015). During moderate sedation, dexmedetomidine decreases global alpha, beta and gamma power, whereas propofol decreases alpha power in the occipital area and increases global beta and gamma power (Akeju et al., 2014). Similarly, in our experiment dexmedetomidine, unlike propofol, reduced pre-stimulus gamma power (Supplementary content: Fig. S3). Our findings, in humans, provide further evidence of the differential roles of dexmedetomidine and propofol on thalamic and cortical oscillations, supporting previous preclinical work (Plourde and Arseneau, 2017; Reed and Plourde, 2015). The novelty of our experiment lies in the demonstration of differences in the task-induced oscillatory changes, with visual-induced GBR being discriminatory between these representative GABA-ergic and non-GABA-ergic sedatives.

#### 4.1.2 Alpha-band responses

Alpha band activity is closely related to the gamma band activity, especially in the occipital cortex (Jensen et al., 2014). While alpha activity is associated with an inhibitory function, in response to a task, it is suppressed to allow high frequency oscillations to transmit information. Thalamo-cortical neurons may be responsible for the generation and maintenance of the alpha-band oscillations (Suffczynski et al., 2001). Modelling studies have suggested that the action of propofol, on these neurones, at unconsciousness producing doses, causes a suppression of posterior alpha and emergence of frontal alpha rhythms (Vijayan et al., 2013). Neural modelling of the changes in the resting EEG spectra during propofol anaesthesia suggest that these are caused by increased inhibition within local interneuron circuits (Ching et al., 2010; Hindriks and van Putten, 2012). An increase in alpha suppression with propofol replicates our previous findings (Saxena et al., 2013), reflecting increased local GABA-ergic inhibitory effects. This increased alpha suppression was not seen with our low contrast visual task. Luminance contrast has been shown to linearly increase the gamma band response and leave beta (13–30 Hz) band response unaffected (Perry et al., 2015). It is unclear if alpha suppression is inversely related to luminance contrast, although there were no differences found between high and low contrast in the placebo group. Our results, however, suggest that propofol induced alpha suppression enhancement may be related to the contrast of the visual stimulus. Dexemdetomidine, does not alter local excitatory-inhibitory balance and therefore demonstrated no effect on task-induced alpha suppression.

### 4.2 Motor oscillatory responses

The results of the motor task related oscillations revealed differences between the two sedatives only in PMBR activity.

#### 4.2.1 MRGS

We had predicted an increase in MRGS with propofol, similar to the increase seen with visual GBR. However, there were no changes in the MRGS activity with either propofol or dexmedetomidine. Interestingly, a similar lack of changes in MRGS with GABA modulators (diazepam (Hall et al., 2011) and tiagabine (GABA transporter inhibitor, which increases synaptic GABA levels) (Muthukumaraswamy et al., 2013)) has been reported. MRGS was enhanced by ketamine (glutamatergic activity) (Shaw et al., 2015) and alcohol (both GABA and glutamatergic activity) (Campbell et al., 2014) suggesting that glutamatergic influence may dominate these oscillations. While we did not directly study glutamatergic drug effects, our findings tend to support previous findings that glutamatergic rather than GABA-ergic effects, may be of greater influence within the excitation-inhibition model influencing the modulation of motor cortical gamma oscillations.

#### 4.2.2 Motor beta-band responses

As a non-specific state of movement preparation, MRBD starts before movement, from the contralateral M1 and then becomes bilateral (Neuper and Pfurtscheller, 2001). Neither dexmedetomidine nor propofol altered contralateral or ipsilateral MRBD. Previously reported increases in MRBD with diazepam (Hall et al., 2011), and tiagabine (Muthukumaraswamy et al., 2013) but not with propofol, suggests that propofol doesoesoes not increase GABA-A activity to the extent required for this effect to be detectable. Unlike, MRBD, the proposed significance of PMBR includes a sensory re-afference to motor cortex following movement (Cassim et al., 2000), stabilising current motor output and, therefore, in preventing initiation of new movements (van Wijk et al., 2012) and reflecting neural processes that evaluate motor error in the context of the prior history of errors (Tan et al., 2014). Propofol showed a non-significant reduction in PMBR (contralateral) while dexmedetomidine increased PMBR (ipsilateral) activity. Interestingly, contrary to other motor beta findings, it has been suggested that PMBR may be a non-GABA-A mediated effect, as evident by absence of effect with diazepam (Hall et al., 2011). Indeed a decrease with tiagabine (Muthukumaraswamy et al., 2013) and propofol (in this experiment)(which has some GABA-B agonist activities (Schwieler et al., 2003)), suggests that this may be a marker of enhanced GABA-B activity. PMBR activity tends to be localised to the contralateral side and the significance on the ipsilateral rebound is less clear. Motor-related tasks which do not involve actual movement such as motor imagery (Pfurtscheller and Neuper, 1997) have shown preferential ipsilateral beta synchrony, while reading (Pfurtscheller, 1992) and movement planning (Pfurtscheller and Neuper, 1994) have shown beta synchrony over both ipsilateral and contralateral M1 areas. Such ipsilateral synchrony may be interpreted as a correlate of a deactivated or actively inhibited motor area neurons wherein enhanced inhibition of the ipsilateral motor area occurs via the transcollosal fibre system (Netz et al., 1995). An increased ipsilateral PMBR with dexmedetomidine, may reflect a more rapid re-afferentation process, which in turn may be a factor in the rapid arousal, as seen clinically, with dexmedetomidine.

### 4.3 Evoked fields (visual and movement-related)

Most anaesthetic drugs affect the amplitudes and latencies of evoked responses in a dose-dependant manner (Kumar et al., 2000). However, at low, equi-sedative doses, dexmedetomdine and propofol had dissimilar effects on the evoked fields. Propofol, but not dexmedetomidine decreased the VEF amplitudes. Both drugs slowed Mv100 but not Mv150. Neither drug had any effect on MF MEF amplitudes, although dexmedetomidine slowed the Mm300.

Visual evoked MEG activity between 80 and 170 ms has been modelled to originate from three sources; one in the calcarine area and two extrastriate sources in dorsal occipito-parietal and ventral occipito-temporal areas, respectively (Supek et al., 1999). The early MEG field response (similar to Mv100 described here) is unaltered by spatial attention, while the later response (similar to Mv150 described here) is modulated by attention. Mv150 VEF have been shown to be localised to the V1 and likely influenced by feedback mechanisms, from higher extrastriate areas (Noesselt et al., 2002). Propofol related suppression of Mv100 and Mv150 amplitudes, similar to Saxena et al. (2013), represents reduced activity of the primary visual cortex and extrastriate cortices, commonly seen in cerebral metabolism /perfusion studies with propofol (Alkire and Haier, 2001; Alkire et al., 1995; Sun et al., 2008). Dexmedetomidine has been shown not to affect evoked responses (including visual); although most studies have been performed during intra-operative use (under anaesthesia with other drugs) (Rozet et al., 2015). We were unable to find any data on dexmedetomidine's independent effects on visual evoked responses. An increased latency of Mv100 with both propofol and dexmedetomidine suggest a comparable degree of thalamocortical delay with both drugs. Our results suggest that at the doses studied in this experiment, dexmedetomdine's cortical effects on VEF are substantially less than that of propofol although effects on thalamocortical conduction may be similar to that of propofol. MEF represent proprioceptive input from the moving digit to the post-central gyrus (Kristeva et al., 1991). While Mm100 likley represents the initial input, Mm300 likely represents a renewed re-afferent input. We can only speculate that the slowing of Mm300 by dexmedetomidine indictates a degree of slowed thalamocortical conduction of this re-afferent input, not seen with propofol.

#### 4.3.1 Limitations

There are some limitations of this study. The visuomotor task employed here is optimised to study the visual oscillations and therefore, the interstimulus interval may not be long enough to capture the entire dynamics of the motor responses, especially the PMBR. While the visuomotor task was designed based on known motor beta band dynamics (Muthukumaraswamy, 2010) and to maintain participant comfort and compliance, especially during sedation, future studies focusing on motor responses to sedative drugs could consider using longer inter-stimulus intervals. Propofol was used as the representative GABA-ergic drug in this study, but due to its additional, albeit minor, effects on other receptors this may have confounded some responses. Future study designs with alternative GABA-ergic sedatives (such as midazolam, which acts only on GABA-A receptors) will further help understand task related motor and visual cortical oscillatory, during sedation.

We conclude that the systems-level effects of GABA-ergic (propofol) and non-GABA-ergic (dexmedetomidine) sedatives are quite distinct, but explainable by their known receptor physiology, and may account for some of the clinical differences observed in their sedative effects. We have shown that at equi-sedative doses, propofol increases visual stimulus-induced GBR while dexmedetomidine decreases it. Dexmedetomidine reduces stimulus-onset-evoked gamma band power, while propofol does not. Propofol reduces VEF (Mv100 and Mv150) amplitudes while dexmedetomidine has no effect on these. Both drugs increased the latency of Mv100 but not the Mv150 VEF. PMBR power is increased by dexmedetomidine while propofol tended to reduce it. Dexmedetomidine also slowed the Mm300 MEF. Better understanding of the neurophysiologic correlates of sedation, based on receptor physiology is likely to help understand the different components of consciousness better, help develop more reliable monitoring tools and help develop anaesthetic drugs with a better safety profile.

## Funding statement

TThe authors acknowledge the generous support of the Wellcome Strategic Award to CUBRIC, ‘Multi-scale and multi-modal assessment of coupling in the healthy and diseased brain’, grant reference 104943/Z/14/Z. This study was funded by National Institute of Academic Anaesthesia, on behalf of AAGBI foundation and supported by the MRC UK MEG Partnership grant (MR/K005464/1) and Wellcome Trust Strategic Award (104,943/Z/14/Z).

## Author contribution

N.S.: study conception, study design, recruitment of volunteers, study conduct, data collection, data analysis, interpretation of results, manuscript preparation.

S.D.M.: study design, recruitment of volunteers, study conduct, data collection, data analysis, interpretation of results, manuscript preparation.

A.B.: recruitment of volunteers, study conduct, data collection.

L.R.: recruitment of volunteers, study conduct, data collection.

K.D.S.: study design, data analysis, interpretation of results and manuscript preparation.

J.E.H.: study design and manuscript preparation.

R.G.W.: study design, interpretation of results and manuscript preparation.

A.D.S.: data analysis, interpretation of results, manuscript preparation.

## Data and code availability statement

De-identified data can be provided by the authors on reasonable request and subject to confidentiality agreements due to ethical constraints.

## CRediT authorship contribution statement

**Neeraj Saxena:** Conceptualization, Visualization, Data curation, Formal analysis, Writing – original draft, Investigation. **Suresh D. Muthukumaraswamy:** Visualization, Data curation, Formal analysis, Writing – original draft, Investigation. **Lewys Richmond:** Visualization, Supervision, Data curation. **Adele Babic:** Visualization, Supervision, Data curation. **Krish D. Singh:** Visualization, Formal analysis, Writing – original draft, Investigation. **Judith E. Hall:** Visualization, Writing – original draft. **Richard G. Wise:** Visualization, Writing – original draft, Investigation. **Alexander D. Shaw:** Formal analysis, Writing – original draft, Investigation.

## CRediT authorship contribution statement

**Neeraj Saxena:** Conceptualization, Visualization, Data curation, Formal analysis, Writing – original draft, Investigation. **Suresh D. Muthukumaraswamy:** Visualization, Data curation, Formal analysis, Writing – original draft, Investigation. **Lewys Richmond:** Visualization, Supervision, Data curation. **Adele Babic:** Visualization, Supervision, Data curation. **Krish D. Singh:** Visualization, Formal analysis, Writing – original draft, Investigation. **Judith E. Hall:** Visualization, Writing – original draft. **Richard G. Wise:** Visualization, Writing – original draft, Investigation. **Alexander D. Shaw:** Formal analysis, Writing – original draft, Investigation.

## Declaration of Competing Interest

None

## Appendix Supplementary materials

Image, application 1
