# Supplementary material for: A comparison of GABA-ergic (propofol) and non-GABA-ergic (dexmedetomidine) sedation on visual and motor cortical oscillations, using magnetoencephalography
Source: Neuroimage. 2021 Dec 15;245:118659. doi: 10.1016/j.neuroimage.2021.118659 (PMC9227747; doi:10.1016/j.neuroimage.2021.118659)
Supplement: Supplementary file 1 [file mmc1.docx]

**Supplementary Content**

**
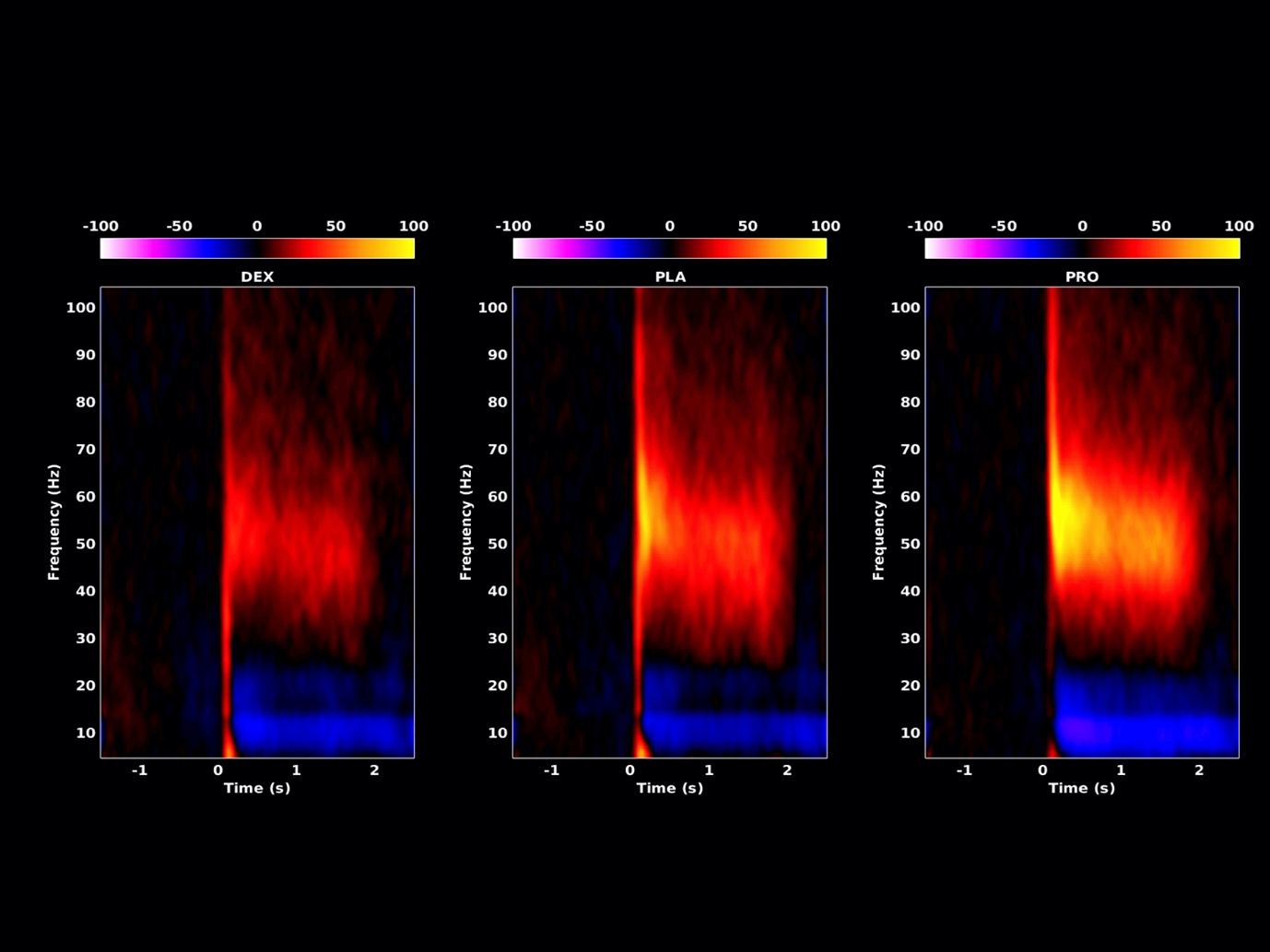
**

Figure S1: Grand-averaged time-frequency spectrograms showing source-level oscillatory amplitude (evoked + induced) changes following visual stimulation with a 70% contrast (low contrast) grating patch (stimulus onset at time = 0) during awake and sedated states. Spectrograms are displayed as percentage change from the pre-stimulus baseline and were computed for frequencies from 5 up to 150 Hz but truncated here to 100 Hz for visualisation purposes. DEX = dexmedetomidine, PLA = placebo, PRO = Propofol.

Results were similar to those obtained with maximum (100%) contrast gratings in the gamma frequency band. In the alpha frequency band there were no group differences.


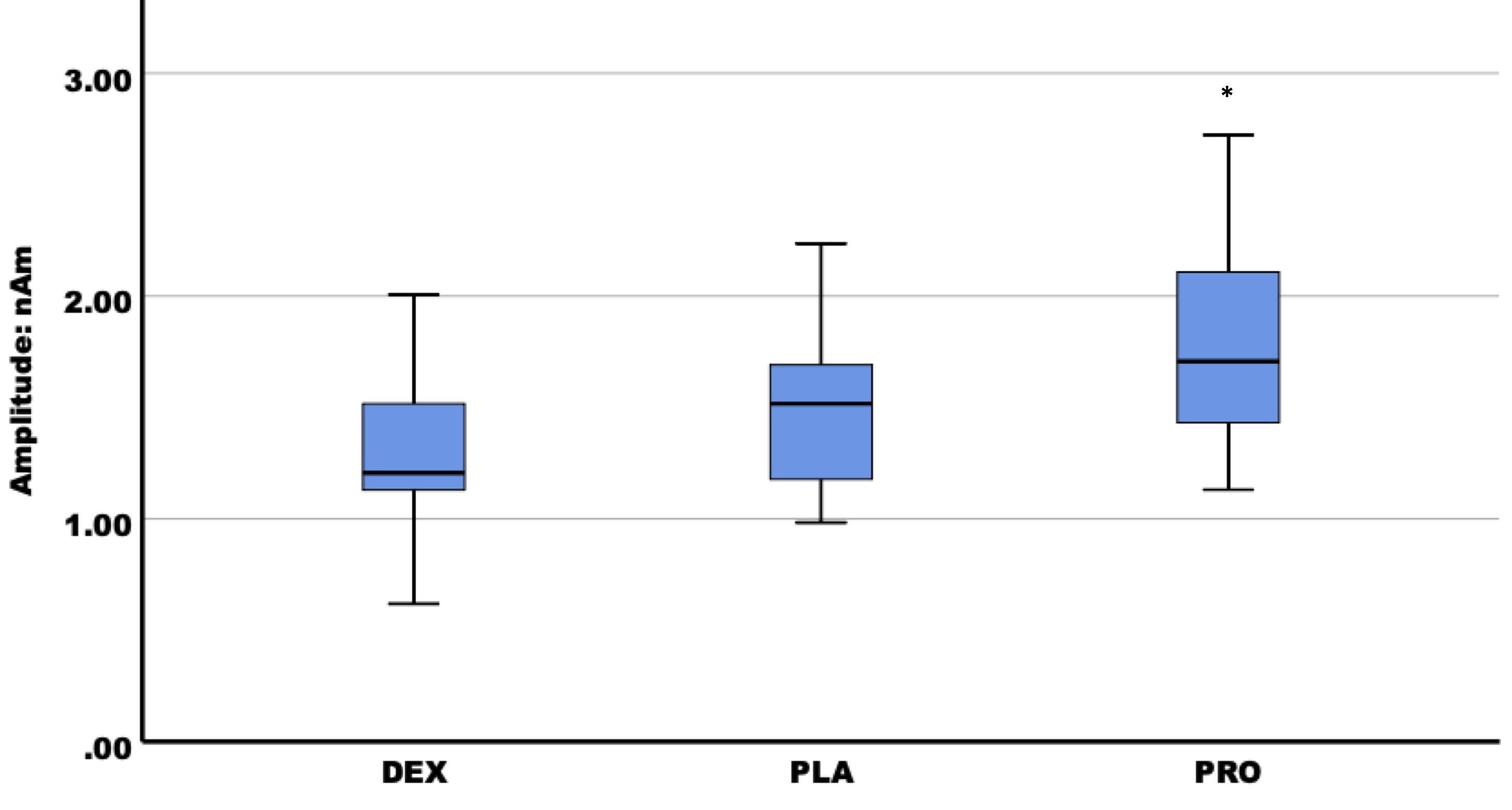


Figure S2: Box plots showing baseline (pre-stimulus) gamma band amplitude changes (high contrast). PLA = placebo, DEX = dexmedetomidine, PRO = propofol. *p < 0.05, compared to placebo.


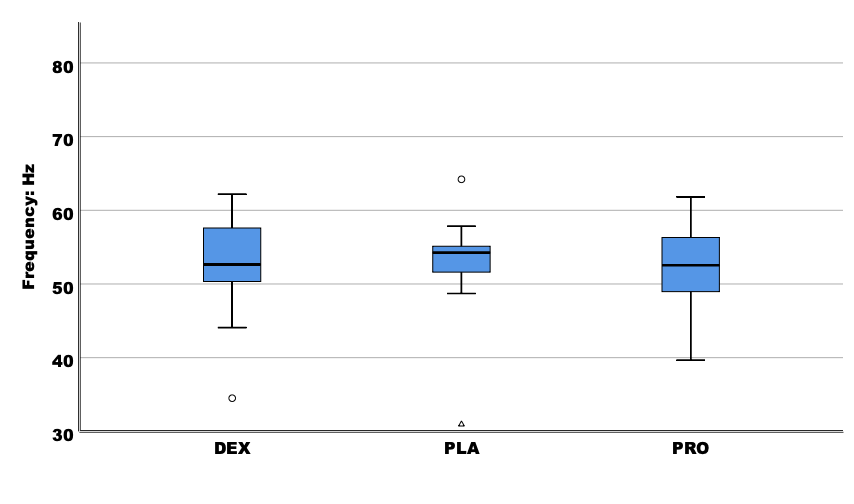


Figure S3: Box plots showing peak frequency in the induced- gamma band (high contrast). PLA = placebo, DEX = dexmedetomidine, PRO = propofol.. There were no significant differences. ^o^ outlier values


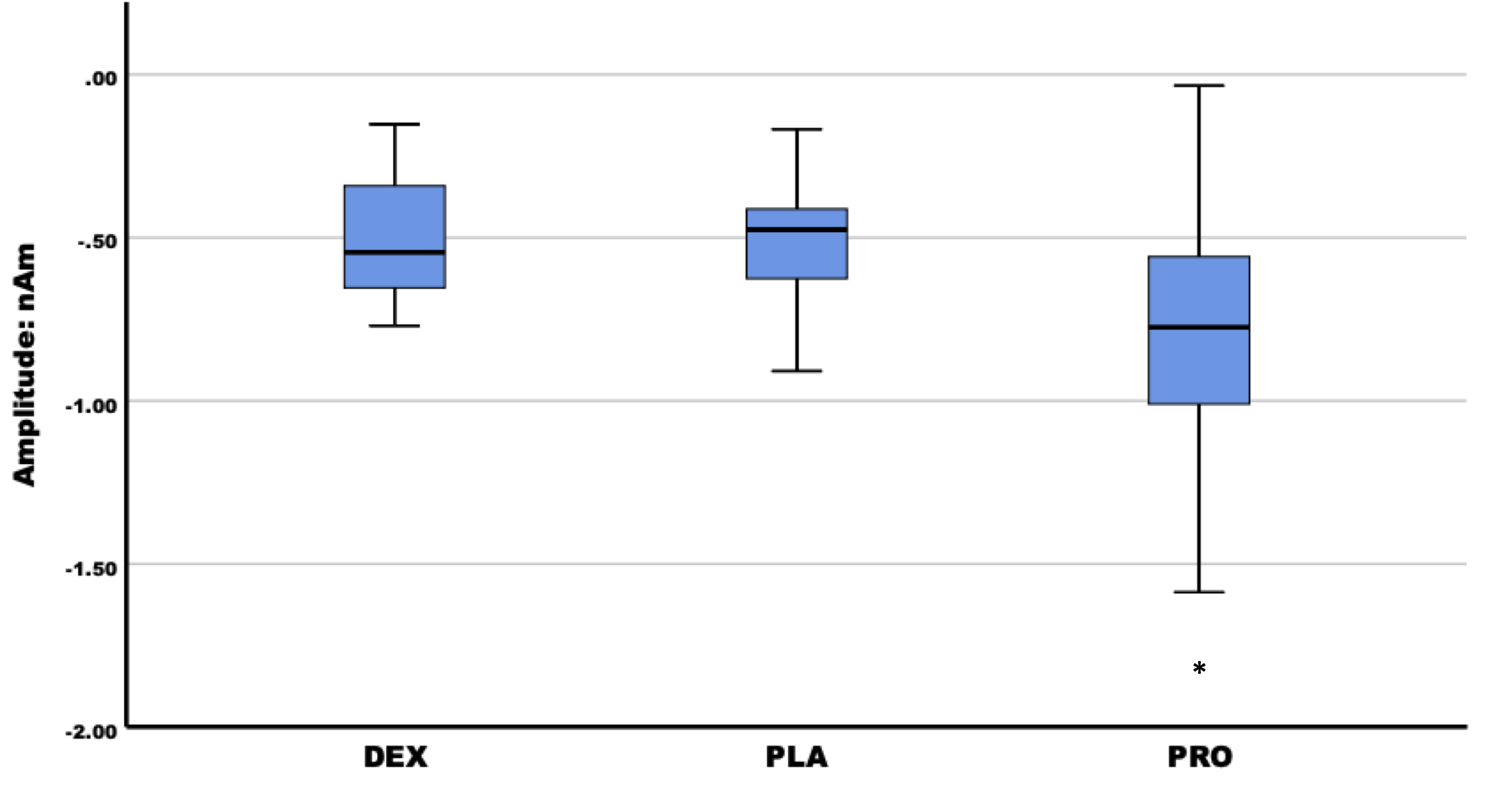


Figure S4: Box-plots showing changes in stimulus induced- alpha band (8-13 Hz) amplitude (change in suppression) during high contrast visual task. PLA = placebo, DEX = dexmedetomidine, PRO = propofol. *p < 0.05, compared to placebo. There were no significant differences during the low contrast visual task.


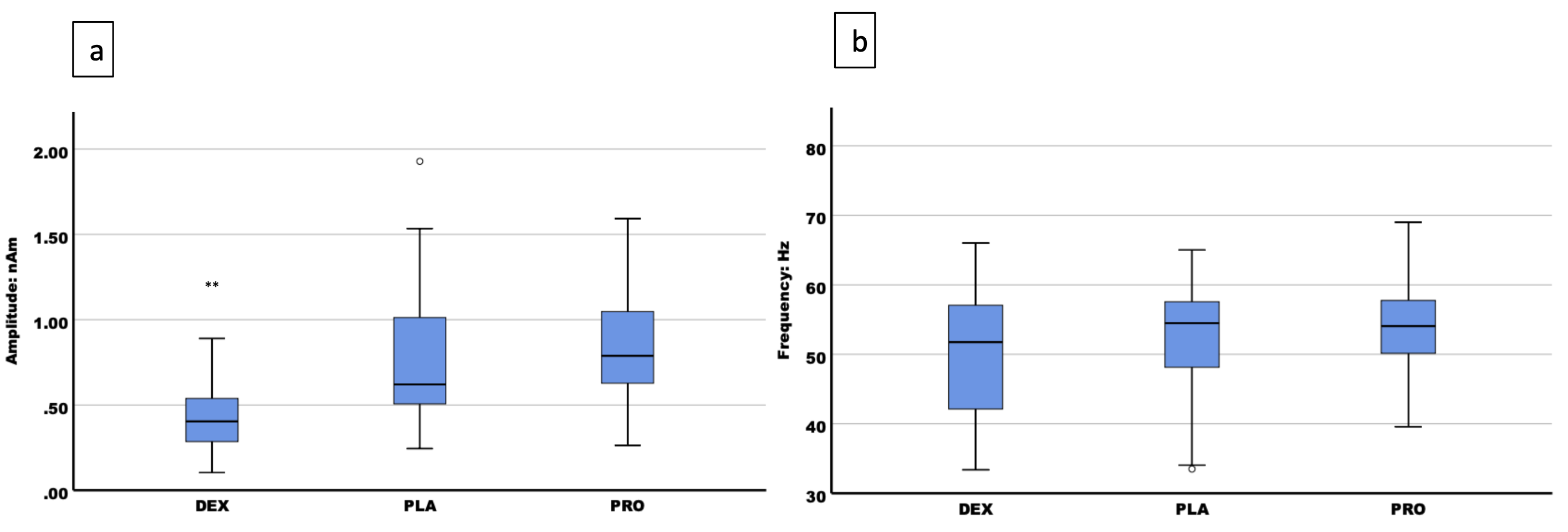


Figure S5: Box-plots of the visual responses during the evoked/transient phase of the high contrast visual task, in the gamma-band range. PLA = placebo, DEX = dexmedetomidine, PRO = propofol. a) amplitude of gamma power , b) peak frequency. (**p < 0.005, compared to placebo. ^o^ outlier values)

**Figure S6:** Box-plots showing source-level time-averaged visual evoked fields for placebo, propofol and dexmedetomidine. PLA = placebo, DEX = dexmedetomidine, PRO = propofol. Significant differences were seen in (a) Mv100 amplitudes (b) Mv100 latency and (c) Mv150 amplitudes (2 tailed paired t-test; difference between drug and placebo: *p < 0.05, ***p < 0.001). No differences were seen in (d) Mv150 latency.

**Table S1. Head movement, reaction times, missed trials and number of trials used in the analyses.**

|  | **Dexmedetomidine** | **Placebo** | **Propofol** | **Statistics** |
| --- | --- | --- | --- | --- |
| **Head movement (mm)** | 3.29 ± 2.9 | 1.57 ± 0.8 | 2.61 ± 3.3 | F (1.3,15.8) = 3.8,  p = 0.058 |
| **Missed trials** | 21.4 ± 8.4* | 4 ± 5.9 | 10.8 ± 11.2 | F (1.09, 11.4) = 6.49;  p = 0.014 ;  *p =0.021 (corr)  paired t test- Dex vs Pla |
| **Reaction times (s)** | 0.32 ± 0.08* | 0.26 ± 0.06 | 0.29 ± 0.09 | F (1.6, 12.9) = 4.69  p = 0.035;  *p =0.008 (corr)  paired t test : Dex vs Pla |
|  | **Trials included in analyses (after rejecting artefacts)** | | | |
| **Visual trials**  **(max 150)** | 118.6 ± 17.1 | 126.6 ± 13.5 | 127.6 ± 19.3 | F (2,30) = 2.59,  p = 0.092 |
| **Motor trials**  **(max 150)** | 115.8 ± 22.3 | 133.4 ± 14.9 | 119.8 ± 29.7 | F (1.5,22.8) = 3.48,  p = 0.059 |
